# Mapping the Relationship between Neighborhood-Level Factors and Nutrition Environments of Restaurants Offering Children’s Menus

**DOI:** 10.1007/s11524-026-01080-4

**Published:** 2026-05-29

**Authors:** Megan Knapp, Maria M. Munoz, Yin Wang, Charles Stoecker, Lisa Hofmann, Melissa Fuster

**Affiliations:** 1 Xavier University of Louisiana, 1 Drexel Dr, New Orleans, LA 70125 USA; 2 Celia Scott Weatherhead School of Public Health and Tropical Medicine, Tulane University, 1440 Canal Street, New Orleans, LA 70112 USA; 3 Department of Social Science and Health Management, College of Public Health, Zhengzhou University, Zhengzhou, China

**Keywords:** Restaurant nutrition environments, Neighborhood nutrition environments, Child nutrition, Child Opportunity Index(COI), Geographic Information Systems (GIS)

## Abstract

This study examined the nutritional environments of restaurants offering children’s menus and their association with neighborhood-level factors in a southern United States city with disproportionately high childhood obesity rates. On-site assessments were conducted to generate nutrition environment scores for restaurants offering children’s menus in New Orleans, Louisiana (*n* = 191). Restaurants were geocoded and linked to neighborhood characteristics, including racial/ethnic composition and socioeconomic opportunity measured by the Social and Economic Child Opportunity Index (COI-SE). Multivariate linear regressions assessed associations between nutrition environment scores and neighborhood characteristics, adjusting for restaurant-specific attributes. Restaurants located in neighborhoods with COI-SEs above the median had significantly higher nutrition environment scores (+ 2.41 points; 95% CI 0.47, 4.35; *p* = 0.015). This relationship appears to be driven by the associations with the healthy options and facilitators to healthy eating sub-scores. Neighborhood racial/ethnic composition was not associated with nutrition environment scores. Restaurant characteristics were associated with nutrition environment quality: sit-down restaurants had lower scores than fast-food restaurants (− 2.35 points; 95% CI −4.45, −0.24; *p* = 0.029), while chain restaurants scored higher than independent establishments (+ 2.13 points; 95% CI 0.44, 3.82; *p* = 0.014). These findings suggest that neighborhood socioeconomic opportunity and restaurant characteristics shape children’s exposure to healthier restaurant food environments. Health strategies that prioritize place-based policies and support healthier children’s menu offerings, particularly in lower-opportunity neighborhoods, may help address inequities in food environments and reduce disparities in child health.

## Introduction

Over the past three decades, childhood obesity rates have continued to increase in the United States (US), posing serious short- and long-term emotional, social, and physical health risks [1–6]. These rates are not evenly distributed across populations; non-Hispanic Black youth and children from lower socioeconomic backgrounds experience disproportionately higher prevalence [7, 8]. Disparities are particularly pronounced in the southern states, where obesity rates are highest. Louisiana, for example, has the third-highest childhood obesity rate nationally (21.8% for 10–17-year-olds) and the fifth-highest adult obesity rate (37.6%) [9], underscoring the urgent need to address place-based drivers of diet-related health inequities.

Diet is a central determinant of obesity and chronic disease risk [10], and children’s dietary behaviors are strongly shaped by their surrounding food environments [11]. Research on community food environments has traditionally emphasized spatial access, such as the availability, proximity, and variety of food outlets [12], yet growing evidence demonstrates that these environments are deeply impacted by neighborhood socioeconomic and demographic characteristics [13–16]. Lower-income and predominantly minoritized neighborhoods are more likely to be characterized by limited access to affordable, healthy foods alongside a high concentration of fast-food outlets and convenience stores offering calorie-dense, nutrient-poor foods and beverages [17–19]. These spatial inequities in food access and nutrition are not incidental but rather reflect longstanding structural and economic processes. Historic practices such as redlining and racially discriminatory housing policies have shaped patterns of neighborhood disinvestment that continue to influence the geographic distribution of food retailers, health-promoting resources, and economic opportunity [20, 21]. In the contemporary context, retail food industry site selection models, driven by purchasing power, perceived risk, and anticipated profitability, reinforce these patterns by disproportionately locating unhealthy food outlets in marginalized communities [21]. As a result, many neighborhoods experience not an absence of food outlets, but an overconcentration of unhealthy options—a phenomenon increasingly described as “food swamps” [22]. For children, inequities in the built and food environments have particularly important implications. Prior studies have shown that neighborhood socioeconomic disadvantage, limited walkability, and reduced access to recreational infrastructure are associated with higher obesity rates among children [10, 13, 23, 24]. Together, these conditions shape children’s everyday dietary exposure and opportunities for healthy behaviors, reinforcing early-life disparities in diet quality and obesity risk.

As dining out becomes increasingly common in the US, restaurants have emerged as a critical component of children’s everyday food environments [11, 25]. Nearly one-third of all calories consumed by Americans comes from food and beverages prepared outside the home, with almost half of food and beverage expenditures spent on dining out [26]. Children are uniquely exposed to restaurant foods through family dining practices, school-adjacent food outlets, and marketing strategies that directly target youth [11, 27]. A substantial literature shows that higher densities of fast-food restaurants around schools and within residential neighborhoods are associated with poorer diet quality and increased obesity risk among children [28]. Research examining restaurant nutrition environments further indicates that children’s menu items are frequently high in calories, sodium, and added sugars, while offering limited fruits, vegetables, and whole grains [29]. Despite this evidence, relatively few studies have explored how neighborhood characteristics shape the nutritional quality of restaurants offering children’s menus.

This paper introduces a novel approach by examining the association between place-based socioeconomic conditions (as measured by the Social Economic Child Opportunity Index [COI-SE]), neighborhood racial and ethnic composition, and nutritional environments of restaurants offering children’s menus. In doing so, the study centers the consumer nutrition environment, defined as the nutritional quality, availability, and marketing of foods as experienced by consumers at the point of purchase [30]. While much food environment research has focused on spatial access, the consumer nutrition environment is particularly salient for understanding children’s dietary exposure in restaurant settings, where menu design, default options, and promotional strategies directly shape consumption [14]. Identifying neighborhood-level factors associated with nutritional environments will help inform targeted, place-based interventions. Furthermore, this approach offers a framework for evaluating the equitable impacts of restaurant-based policies, such as Healthy Default Beverages (HDB) policies. These efforts are essential to understanding and addressing the unequal distribution of diet-related health conditions and promoting healthier food environments for children.

## Methods

### Study Design and Setting

This cross-sectional study was conducted in New Orleans, Louisiana, as part of a larger, ongoing nutrition policy evaluation project. New Orleans is known for its food and beverage offerings but also for the pressing health issues. The city’s demographics are largely black or African American, with above average poverty rate of 23% (National rate: 10.6%) [31]. About two-thirds of the NO population is overweight or obese and 12% have diabetes, with rates higher among minorities and those with lower income [32, 33]. Data collection focused on restaurants that offered children’s menus to assess the nutrition environment.

### Sample Development

A list of 1108 permitted food operators was obtained from the City of New Orleans website. The list was filtered by business type to include restaurants, catering services, and food preparation establishments. Eligible businesses met the following criteria: operated as a restaurant open to the general public; offered a children’s menu; and were located within Orleans Parish.

Trained research assistants (RAs) conducted an initial online screening to verify the availability of a children’s menu at each restaurant. If a children’s menu could not be confirmed online, RAs contacted restaurants via phone, email, or social media. Restaurants that did not offer a children’s menu or were not open to the general public (e.g., restaurants located within museums) were excluded due to access feasibility for the assessment team.

For chain restaurants with more than five locations in New Orleans, five sites were randomly selected for on-site assessments to ensure a more balanced sample of independently owned and corporate restaurants. This sampling approach was used for the original study from which these data were drawn, as part of a baseline assessment of menus conducted prior to implementation of the New Orleans Healthy Kids Menu Ordinance [34]. Characteristics and scores from the five assessed locations were averaged and assigned to the remaining locations within the same chain.

On-site assessments were conducted at 139 restaurants representing 102 unique restaurant brands. After accounting for additional eligible chain locations, a total of 191 restaurants were included in the final analytic sample.

### Data Collection

Pairs of trained research assistants conducted on-site visits to assess the nutrition environment. During each visit, they documented restaurant characteristics and photographed menus for subsequent off-site analysis using a paper-based assessment tool adapted from the Nutrition Environment Measures Survey for Restaurants (NEMS-R). Additional items were drawn from the Children’s Menu Assessment (CMA), an expansion of NEMS-R, to more comprehensively capture children’s menu characteristics, including healthy side offerings [35–37]. NEMS-R assesses the healthfulness of foods and beverages offered on main and children’s menus, with emphasis on availability, facilitators and barriers to healthful eating, pricing, and signage or promotion. The assessment tool is comprised of five domains: (1) healthy options, (2) children’s menu, (3) facilitators to healthy eating, (4) barriers to healthy eating, and (5) pricing [35]. Items from the CMA were incorporated to provide additional detail on children’s menu side dishes [36]. Full details of the assessment tool are provided in Supplement 1.

During visits, RAs recorded restaurant types, categorized as fast food (emphasizing speed and convenience), fast casual (counter-service with higher quality food), or sit-down (full waiter service). They also documented menu offerings, promotional signage, and accessibility. The midpoint price of main dishes was calculated as the difference between the highest and lowest priced main dish, divided by two.

### Menu Scoring and Quality Control

A separate team of trained RAs reviewed the menu photos and applied the modified environmental assessment tools to evaluate the nutritional quality of each restaurant’s offerings. A senior researcher reviewed all assessments to ensure scoring accuracy and consistency.

Restaurant nutrition environment scores were calculated using adapted NEMS-R scoring protocols with additional points integrated for the CMA items [35, 36]. Each restaurant received an overall score based on the sum of domain-specific sub-scores. Final scores ranged from −14 to +38, with higher scores indicating healthier food environment and menu offerings. Sub-score ranges were as follows: healthy options (−1 to 18), kid’s menu (−5 to 12), facilitator (0 to 6), barriers (−5 to 0), and pricing (−3 to 2).

### Neighborhood Characteristics

Restaurant addresses were geocoded using a geographic information system (QGIS version 3.16) and verified through their websites and Google Maps. To delineate the service area for each restaurant, 800-m network buffers were constructed along New Orleans, LA street network. Unlike straight-line Euclidean buffers, these network buffers account for real-world barriers, such as rivers and private land, providing a more accurate representation of neighborhoods. The 800-m distance represents a convenient 10–15 min’ walking radius for food access [38, 39].

Within each service area (SA), we estimated population-weighted census tract (CT)-level sociodemographic characteristics to describe a restaurant’s spatial context. To improve the accuracy of weight measures, we leveraged the populations of block groups (BGs), a finer geographic unit nested within a CT, to obtain the population of a BG-SA intersected area using proportional areal weighting. This population was then aggregated to derive population estimates for each CT-SA intersected area as well as for the entire SA. The proportion of population contributed by each CT-SA intersected area relative to the total SA population was used as weights to compute weighted averages of CT-level socioeconomic measures, including COI-SE scores, racial composition, and age group proportions, for each SA. The CT-level demographic measures and BG-level population data were obtained from the American Community Survey 2018–2022 5-year estimates [40]. The New Orleans street network, along with BG and CT boundaries, was sourced from Topologically Integrated Geographic Encoding and Referencing (TIGER)/Line Shapefiles [41]. The CT-level COI-SE scores were sourced from the COI 3.0 database [42]. The COI-SE score includes values from 19 indicators that are predictive of children’s short- and long-term developmental outcomes. These indicators relate to employment, economic resources, socioeconomic inequity, housing resources, social resources, and wealth. Scores range from 1 (lowest opportunity) to 100 (highest opportunity).

### Statistical Analysis

We employed multiple approaches to examine the interplay between the restaurant nutrition environment and neighborhood characteristics. Initially, maps were created using QGIS 3.16 software to visualize the geographic distribution of sampled restaurants, along with total nutrition environment scores and neighborhood sociodemographic attributes. Descriptive statistics were further computed for the full sample and stratified by neighborhoods based on racial composition and child opportunity index. Restaurants in neighborhoods with predominately visible minority residents (non-White % ≥ 60%) were compared to those with lower visible minority residents (non-White % < 60%) [43], and restaurants in neighborhoods with above-median COI-SE (more opportunities) were compared to those with below median COI-SE. Lastly, we performed multivariate linear regression analysis by STATA 16.0 software to evaluate the associations between restaurants’ nutrition environment scores and the characteristics of their surrounding neighborhoods (COI-SE, visible minority, and age group proportions), adjusting for restaurant-specific attributes. Estimates with *p* < 0.05 were considered statistically significant.

## Results

Our study sample consisted of 191 restaurants with children’s menus across New Orleans. Of the restaurants evaluated, the majority were categorized as either sit-down establishments (47.1%) or fast-food outlets (41.9%). A substantial proportion (66.5%) belonged to a restaurant chain (Table 1). The mean midpoint price of a main dish across all restaurants was $12.36. The average total nutrition environment score was 4.37 (SD = 4.57), with observed values ranging from −3 to 17. Mean sub-scores by domain were as follows: healthy options, 2.4 (SD = 2.83); children’s menu, 1.78 (SD = 1.63); facilitators of healthy eating, 1.15 (SD = 1.16); barriers to healthy eating, −0.88 (SD = 0.99); and pricing, −0.08 (SD = 0.30).

**Table 1:** Descriptive statistics of full restaurant sample by racial composition and by neighborhood opportunity

	Full sample		Predominantly visible minority neighborhoods^a^		Neighborhoods with COI-SE scores ≥ median	
Restaurant characteristics						
	***N***	**%**	***N***	**%**	***N***	**%**
Fast food restaurants	80	41.9	49	65.3	10	10.4
Fast casual restaurants	21	11.0	9	12.0	23	24.0
Sit-down restaurants	90	47.1	17	22.7	63	65.6
Independent restaurants	64	33.5	13	17.3	46	47.9
Chain restaurants	127	66.5	62	82.7	50	52.1
*N*	191		75		96	
	**Mean**	**SD**	**Mean**	**SD**	**Mean**	**SD**
Adult main dish midpoint price^b^ ($)	12.36	7.82	8.94	6.03	14.53	8.07
**Restaurant nutrition environment**						
Total score	4.37	4.67	4.54	4.75	4.56	4.61
Children’s menu sub-score	1.78	1.63	1.98	1.73	1.70	1.61
Healthy options sub-score	2.41	2.82	2.49	2.73	2.49	2.86
Facilitators to healthy eating sub-score	1.15	1.16	1.32	1.06	1.09	1.26
Barriers to healthy eating sub-score	− 0.88	0.99	− 1.21	1.12	− 0.61	0.83
Pricing sub-score	− 0.08	0.30	− 0.04	0.24	− 0.12	0.35

*Notes: COI-SE* child opportunity index for the socioeconomic domain, *SD* standard deviation

^a^Neighborhoods with visible minority population % ≥ 60%

^b^There were two restaurants with no data for adult main dish midpoint price

Descriptive statistics revealed variations in nutrition environments across neighborhoods categorized by racial composition and child opportunity. Overall, fewer restaurants (75 out of 191) were in predominantly visible minority neighborhoods. Predominantly visible minority neighborhoods had a higher concentration of fast-food restaurants (65.3%) compared to the full sample (41.9%), while neighborhoods with above-median COI-SE scores had a greater prevalence of sit-down restaurants (65.6%). Mean midpoint prices for adult main dishes were notably lower in predominantly visible minority neighborhoods ($8.94) and higher in neighborhoods with above-median COI-SE scores ($14.53) compared to the full sample.

Although total nutrition environment scores varied only slightly across neighborhood types (ranging from 4.26 to 4.56), sub-scores for barriers to healthy eating were higher in predominantly visible minority neighborhoods (–1.21) compared to the full sample (–0.88) and neighborhoods with above-median COI-SE scores (–0.61), with greater variability observed (SD = 1.12). These findings suggest disparities in both nutrition environment and restaurant characteristics.

**Fig. 1:**
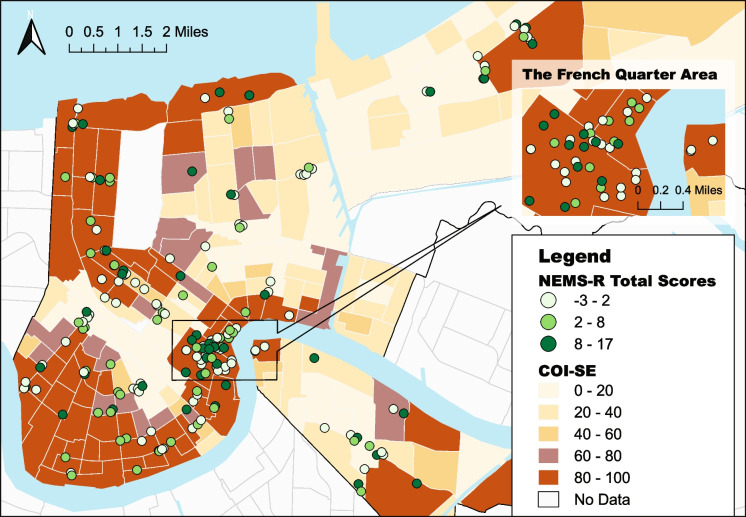

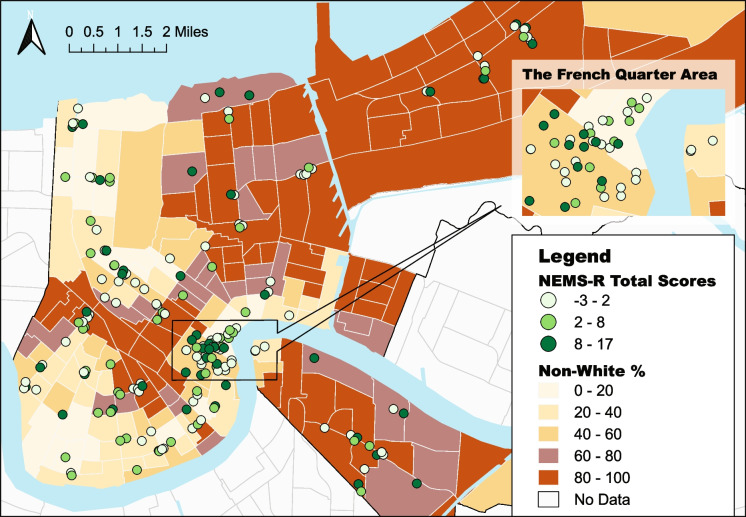
Distribution of restaurants (*N*** = **191) and sociodemographic attributes of census tracts

Figure 1 illustrates the geographic distribution of these restaurants over census tracts shaded by socioeconomic child opportunity index (COI-SE) scores and the proportion of the visible minority population. While Fig. 1 provides a spatial overview, it does not demonstrate clear relationships between restaurants’ total nutrition environment scores and census tract sociodemographic characteristics, as restaurants with varying nutrition environments were often located in close proximity. The French Quarter, a prominent tourist destination in New Orleans, emerged as a cluster with mixed nutrition environment scores, ranging from very healthy to negative scores.

### Factors Associated with Total Nutrition Environment Scores

Regression analyses were employed to examine the association between a restaurant’s total nutrition environment score and its neighborhood characteristics regarding socioeconomic opportunity, racial composition, and population age structure (Table 2). The findings suggest a positive association between total nutrition environment scores and socioeconomic opportunity, as measured by the COI-SE score. Specifically, after adjusting for potential confounders, restaurants located in neighborhoods with COI-SE scores above the median were associated with a statistically significant increase of 2.413 points (95% CI 0.474 to 4.352; *P* = 0.015) in total nutrition environment scores compared to those in neighborhoods with below-median COI-SE scores. No significant associations were observed between total scores and the presence in predominantly visible minority neighborhoods or the proportion of non-adults in the surrounding population. Furthermore, restaurant service type and chain affiliation influenced scores: sit-down restaurants had lower scores (a decrease of 2.347 points, 95% CI −4.453 to −0.240; *P* = 0.029) compared to fast-food restaurants, while chain restaurants scored 2.131 points higher (95% CI 0.439 to 3.822; *P* = 0.014) than independent establishments. These results highlight the influence of both neighborhood socioeconomic opportunity and restaurant characteristics on the quality of the consumer nutrition environment.

**Table 2:** Associations of total nutrition environment scores with neighborhood and restaurant characteristics

	**Total nutrition environment scores**			
	(1)	(2)	(3)	(4)
**Neighborhood characteristics**				
COI-SE score ≥ median (Ref: < median)	0.379	1.313	1.443	2.413*
	[− 0.957, 1.715]	[− 0.684, 3.309]	[− 0.588, 3.473]	[0.474, 4.352]
Visible minority % ≥ 60% (Ref: < 60%)		1.285	1.004	− 0.168
		[− 0.760, 3.329]	[− 1.181, 3.189]	[− 2.250, 1.915]
0–18 years %			0.044	0.062
			[− 0.076, 0.165]	[− 0.053, 0.176]
**Restaurant characteristics**				
Fast casual restaurants (Ref: fast food restaurants)				− 1.134
				[− 3.534, 1.265]
Sit-down restaurants (Ref: fast food restaurants)				− 2.347*
				[− 4.453, − 0.240]
Restaurants in-chains (Ref: independent restaurants)				2.131*
				[0.439, 3.822]
Adult main dish midpoint price^a^				− 0.044
				[− 0.166, 0.078]
*N*	191	191	191	189

Notes: Each column is a separate regression where the outcome is the total nutrition environment score of each restaurant

*Ref* reference, which denotes the reference category of the regression estimates for categorical variables, *COI-SE* child opportunity index for the socioeconomic domain

^*^p < 0.05, **p < 0.01

^a^There were two restaurants with no data for adult main dish midpoint price

#### Factors Associated with Nutrition Environment Sub-scores Across Five Domains

Sub-scores analysis confirmed that restaurants in neighborhoods with above-median COI–SE scores had significantly higher sub-scores in the healthy options domain (1.243 point increase, 95% CI 0.062 to 2.424; *P* = 0.048) and the facilitators to healthy eating domain (0.464 point increase, 95% CI 0.004 to 0.925; *P* = 0.039) compared to those in neighborhoods with lower COI–SE scores. However, no statistically significant associations were observed for the kid’s menu, barriers to healthy eating, or pricing sub-scores with respect to neighborhood COI–SE scores or other demographics (Table 3).

**Table 3:** Associations of nutrition environment sub-scores with neighborhood and restaurant characteristics

	**Kid’s menu sub-score**	**Healthy options sub-score**	**Facilitators to healthy eating sub-score**	**Barriers to healthy eating sub-score**	**Pricing sub-score**
**Neighborhood characteristics**					
COI-SE score ≥ median (Ref: < median)	0.611	1.243*	0.464*	0.171	− 0.077
	[− 0.039, 1.261]	[0.062, 2.424]	[0.004, 0.925]	[− 0.191, 0.533]	[− 0.205, 0.052]
Visible minority % ≥ 60% (Ref: < 60%)	− 0.041	− 0.228	0.09	− 0.007	0.019
	[− 0.739, 0.656]	[− 1.497, 1.040]	[− 0.405, 0.584]	[− 0.396, 0.382]	[− 0.119, 0.157]
0–18 years %	0.015	0.039	0.003	0.008	− 0.002
	[− 0.024, 0.053]	[− 0.031, 0.109]	[− 0.025, 0.030]	[− 0.014, 0.029]	[− 0.010, 0.005]
**Restaurant characteristics**					
Fast casual restaurants (Ref: fast food restaurants)	− 1.039*	− 0.208	− 0.645*	0.917**	− 0.159*
	[− 1.844, − 0.235]	[− 1.669, 1.253]	[− 1.214, − 0.075]	[0.469, 1.365]	[− 0.319, − 0.000]
Sit-down restaurants (Ref: fast food restaurants)	− 1.478**	− 0.77	− 0.854**	0.962**	− 0.206**
	[− 2.184, − 0.772]	[− 2.053, 0.513]	[− 1.354, − 0.354]	[0.569, 1.355]	[− 0.346, − 0.067]
Restaurants in-chains (Ref: independent restaurants)	0.603*	1.541**	0.384	− 0.16	− 0.237**
	[0.036, 1.169]	[0.511, 2.572]	[− 0.018, 0.786]	[− 0.476, 0.156]	[− 0.349, − 0.125]
Adult main dish midpoint price^a^	0.004	− 0.043	− 0.015	0.009	0.001
	[− 0.037, 0.045]	[− 0.118, 0.031]	[− 0.044, 0.014]	[− 0.013, 0.032]	[− 0.007, 0.009]
*N*	189	189	189	189	189

Notes: Each column is a separate regression where the outcome is indicated in the first row

*Ref* reference, which denotes the reference category of the regression estimates for categorical variables, *COI-SE* child opportunity index for the socioeconomic domain

^*^p < 0.05, **p < 0.01

Furthermore, compared to fast food restaurants, fast casual and sit-down establishments scored significantly lower in the nutritional quality of kids’ menus, facilitators to healthy eating, and pricing domains (reductions ranging from −0.159 to −1.478 points) but higher in the barriers to healthy eating domain by 0.9 points. Chain restaurants outperformed independent restaurants in the kids’ menu domain (0.603 point increase, 95% CI 0.036 to 1.169; *P* = 0.037) and the healthy options domain (1.541 point increase, 95% CI 0.511 to 2.572; *P* = 0.004), though they scored slightly lower in the pricing domain (0.237 point decrease, 95% CI −0.349 to −0.125; *P* < 0.001).

## Discussion

This study examined the nutritional quality of restaurants with children’s menus in New Orleans, Louisiana, and their associations with neighborhood socioeconomic and racial/ethnic characteristics by applying COI-SE data, census tract data, and detailed restaurant environment assessments. The findings underscore the importance of neighborhood socioeconomic opportunity in shaping the nutritional environment, revealing both opportunities for targeted interventions and highlighting persistent gaps.

Consistent with previous research, our results demonstrate a positive association between neighborhoods with greater socioeconomic opportunity, as measured by COI-SE, and healthier restaurant nutrition environments [44–46]. Restaurants in neighborhoods with above-median COI-SE scores had higher total nutrition environment scores, suggesting that children in these areas may have better access to more nutritious foods when dining out. Moreover, a higher number of restaurants were in areas with predominantly White populations and greater socioeconomic opportunity, highlighting persistent inequities in food access [14, 18]. Addressing these disparities will require more than improving socioeconomic conditions alone; targeted efforts to address systemic barriers and promote equity in food access are also critical. For example, policies that incentivize the establishment of food outlets in underserved neighborhoods help reduce these disparities.

Although restaurants in predominantly minority neighborhoods faced more barriers to healthy eating, overall nutrition environment scores did not vary significantly across neighborhoods with different racial compositions, consistent with findings from previous research [45]. This lack of variation may be influenced by contextual factors specific to New Orleans, such as its strong tourism economy and the high proportion of Black residents (57%) [46]. While socioeconomic conditions appear to play a significant role in shaping healthy menu offerings, racial differences may be more evident in the types and availability of restaurants. These results align with prior studies suggesting that socioeconomic and ethnic and racial composition can independently affect neighborhood food environments [36, 43].

Another notable finding is the impact of restaurant characteristics on nutrition environment scores. Chain restaurants outperformed independent establishments, particularly in the healthy options and kid’s menu offerings domains. This may be the result of corporate level decisions or the influence of policies like Sect. 4205 of the 2010 Affordable Care Act, Public Law 111–148 (HR 3590) that mandates restaurant chains with 20 or more US locations disclose calories to customers [47]. In contrast, sit-down and fast-casual restaurants exhibited lower scores, especially in domains related to kid’s menus and facilitators to healthy eating. These results indicate a need to engage a broad range of stakeholders in nutrition policy efforts and interventions that target independent, sit-down, and fast-casual restaurants, which may lack the regulation, resources, or incentives to prioritize healthier offerings. Interventions such as HDB initiatives or nutrition standards for children’s menus may be particularly relevant for these types of establishments. These interventions could help standardize offerings and reduce variability in the quality of children’s menus. Future research should evaluate the effectiveness of such interventions, with a focus on their impact in neighborhoods with varying levels of socioeconomic opportunity.

This study has several strengths, including its use of geospatial analyses to contextualize neighborhood characteristics, a comprehensive assessment of restaurant nutrition environments, and the novel use of COI. However, it also has limitations. One limitation is that, due to its observational design, the identified associations cannot be interpreted as evidence of causation. Second, the study’s focus on New Orleans, Louisiana, may limit the generalizability of its findings to cities in other regions. Third, while the COI–SE provides a measure of neighborhood opportunity, it does not capture all dimensions of the built environment that may influence food access and dietary behaviors. Future studies may consider incorporating additional measures, such as individual-level dietary data, to build on these findings.

In conclusion, this study highlights the critical role of neighborhood socioeconomic opportunity and restaurant characteristics in shaping the nutrition environment for children. While higher-opportunity neighborhoods tend to offer healthier restaurant environments and options, disparities in predominantly visible minority neighborhoods and among independent or sit-down restaurants point to persistent inequities in food access. Addressing these issues will require targeted, multilevel interventions that prioritize equity and engage diverse stakeholders. Improving restaurant nutrition environments and ensuring equitable access to nutritious foods could play a vital role in preventing childhood obesity and fostering healthier dietary habits among children.

## Data Availability

The data that support the findings of this study are available from the corresponding author upon reasonable request.
